# Microalgae Harvesting after Tertiary Wastewater Treatment with White-Rot Fungi

**DOI:** 10.3390/jof8111232

**Published:** 2022-11-21

**Authors:** Anna Civzele, Linda Mezule

**Affiliations:** Water Research and Environmental Biotechnology Laboratory, Water Systems and Biotechnology Institute, Faculty of Civil Engineering, Riga Technical University, LV-1048 Riga, Latvia

**Keywords:** microalgae harvesting, *Irpex lacteus*, filamentous fungi, bio-flocculation, wastewater treatment, white-rot fungi

## Abstract

Tertiary wastewater treatment with microalgae incorporates environmental sustainability with future technologies and high exploitation costs. Despite the apparent ecological benefits of microalgae-assisted wastewater treatment/biomass-based resource production, technological improvements are still essential to compete with other technologies. Bio-flocculation instead of mechanical harvesting has been demonstrated as an alternative cost-effective approach. So far, mostly filamentous fungi of genus *Aspergillus* have been used for this purpose. Within this study, we demonstrate a novel approach of using white-rot fungi, with especially high potential of algae—*Irpex lacteus* complex that demonstrates efficiency with various microalgae species at a broad range of temperatures (5–20 °C) and various pH levels. Harvesting of microalgae from primary and secondary wastewater resulted in 73–93% removal efficiencies within the first 24 h and up to 95% after 48 h. The apparent reuse potential of the algae—*I. lacteus* pellets further complements the reduced operating costs and environmental sustainability of bio-flocculation technology.

## 1. Introduction

Microalgae cultivation and biomass exploitation has been widely studied, and technology scale-up to an industrial level is well known. Moreover, the potential of microalgae has been also recognized by non-fuel industries, including wastewater treatment [1,2,3,4], where the cultivation of the algae not only produces valuable biomass, but also effectively removes excess nutrients. Introduction of microalgae in wastewater treatment can increase the overall nitrogen and phosphorous removal efficiency from 39 to 66% and from 32 to 89% [5], respectively, thus minimizing the impact of wastewater on the eutrophication of natural waters [6].

Still, one of the major technological bottlenecks in the wide scale application of microalgae for tertiary wastewater treatment is related to biomass harvesting, which can contribute up to 30% of the total production costs, mainly due to the small size of the microalgae cells (5–30 µm) [7] and their colloidal stability in suspension [8]. One of the simplest harvesting approaches is sedimentation which is known for low capital and operating costs, low energy demand and no disruptive effect on the cells. However, it requires high land area [7], leaving no possibility to be introduced into most of the wastewater treatment plants (WWTPs). Alternatively, physical (filtration, gravity sedimentation, flotation and centrifugation), chemical (chemical coagulation and flocculation), biological (bio-flocculation), electrical or a combination of these harvesting methods [9] can be used. Flotation and sedimentation are economically feasible and simple, but they usually require prior chemical coagulation or flocculation [10,11,12]. These technologies in turn can be fast and simple, but toxic to the microalgae and also expensive due to the requirements of chemicals [8]. Electrical methods, on the other hand, do not require the use of chemicals [13], but have high operating costs due to high electricity consumption [14]. Filtration and centrifugation are also characterized by high operational and capital costs, but these methods have high recovery efficiencies [15,16].

In recent years, bio-flocculation using other microorganisms has been offered as a method for microalgae harvesting [9,17,18,19]. The process of microalgae bio-flocculation with filamentous fungi of the genus *Aspergillus* is the most widely described [20,21,22,23,24,25]. By employing pellet-assisted harvesting, more than 90% removal efficiency of *Chlorella vulgaris* was achieved after 24 h due to bio-flocculation caused by *Aspergillus niger* [20]. With *Aspergillus fumigatus*, the concentration of *Scenedesmus quadricauda* was reduced by more than 95% in 48 h [21] and *Chlorella protothecoides* by 80% in 24 h [22]. Integrating spore-assisted harvesting, 93% and more than 95% removal efficiency of *Chlorella vulgaris* was provided by adding *Aspergillus oryzae* [23] and *A. niger* [24], respectively, in 72 h. It was also reported that 98% *Botryococcus braunii* harvesting efficiency was ensured within 12 h by adding *A. fumigatus* [25]. Furthermore, when the biomass flocculation occurs, it can be removed with traditional sedimentation, non-reagent flotation or sieving. Given the non-toxicity of this technology in general, the potential cost-effectiveness and the high-harvesting efficiency [9,26], microalgae co-cultivation with filamentous fungi can be considered as a potentially efficient and optimal method of microalgae harvesting. It was also reported that the combination of fungal and algal biomass may cause positive effects on the total biofuel production, and harvested fungi-algae pellets could be considered as value-added feedstock for the production of biofuels, including biodiesel, biomethane, bioethanol and biohydrogen [9]. However, despite the well-demonstrated efficiency of this technology [20,21,22,23,24,25], it is still blanked out by a lack of large-scale testing, relatively long flocculation times and limitations in the reuse of the obtained biomass after wastewater treatment and possible wastewater contamination with *Aspergillus* spp. spores and its leakage into the environment.

In this study, an alternative microalgae bio-flocculation technology using white-rot and soil fungi was investigated. The potential of using filamentous white-rot fungi such as *Irpex lacteus*, *Trametes versicolor*, *Pleurotus ostreatus* and soil fungi such as *Trichoderma reesei* has been demonstrated in the degradation of pharmaceutical substances from wastewater [27] and lignocellulosic biomass with high enzymatic activity in the apex of the hyphae [28,29]. Based on these investigations and the observed extensive formation of hyphae, it has been proposed that these fungi may be suitable candidates for microalgae harvesting from wastewater and subsequent lignocellulose-degrading enzyme production to improve the hydrolysis process of cellulosic materials that are essential for bioethanol production [30]. Moreover, considering the lower risk of the aforementioned fungi affecting human health compared to *Aspergillus* spp. [31,32,33], the use of white-rot fungi could become a safe alternative to pathogenic fungal species in the microalgae removal process. Furthermore, these experimental studies have suggested the most suitable cultivation and handling conditions and demonstrate the reuse of the material during the harvesting process, thus, limiting the amount of process waste.

## 2. Materials and Methods

### 2.1. Microorganisms and Culture Conditions

*Tetradesmus obliquus* (CCAP 276/10), *Desmodesmus communis* (CCAP 276/4B) and *Chlorella vulgaris* (CCAP 211/11B) were used as representative microalgae within this study. For harvesting, microalgae suspension was prepared by culturing the microalgae in 1000 mL Pyrex^®^ bottles in BG-11 growth medium for 10 days at 20–27 °C. Continuous 10 Lh^−1^ aeration and the blue-red spectrum fluorescent light (180 μmol m^2^ s^−1^ at a 16:8 h lighting regime) was provided during microalgae cultivation. Microalgae cells for microalgae harvesting tests were obtained during the exponential phase.

White-rot fungi *Irpex lacteus* (Fr.) Fr., *Pleurotus dryinus* (Pers.) P. Kumm, *Pleurotus ostreatus* (DSM 1020), *Trametes versicolor* (DSM 6401), *Pycnoporus cinnabarinus* (Fr.) P. Karst and soil fungus *Trichoderma reesei* (DSM 768) were maintained on potato dextrose agar (Oxoid Ltd., Basingstoke, Hants, UK) at 2–8 °C. Each fungal species was inoculated into 250 mL Erlenmeyer flasks in a culture medium containing 0.8 g KH_2_PO_4_, 0.4 g K_2_HPO_4_, 0.5 g MgSO_4_·7H_2_O, 2 g NH_4_NO_3_, 2 g yeast extract and 10 g glucose per L and then cultivated for 3 days in an orbital shaker (New Brunswick™ Innova^®^ 43, Eppendorf Austria GmbH, Wien, Austria) at 150 rpm and 30 °C. The pH level in the medium was adjusted to 5.3–5.5.

### 2.2. Wastewater Source

Primary and secondary effluents were collected at a biological wastewater treatment plant “Daugavgriva” (Riga, Latvia, PE > 100 000) after primary settlers and after biochemical oxidation and secondary settlers. Prior to use, the wastewater was filtered through a 0.45 µm cellulose-acetate filter to remove indigenous bacteria and microparticles. The concentrations of all quality parameters for both types of wastewater (Table 1) were provided using internal WWTP monitoring performed according to standard methodology [34,35,36,37,38,39,40,41].

### 2.3. Experimental Setup

Pre-cultured fungal pellets were mixed with a heat-pretreated microalgae culture with a concentration of 5 × 10^6^ cell/mL in Schott Duran 100 mL laboratory bottles, which were then placed on an orbital shaker (PSU-20i, Biosan, Riga, Latvia) at 150 rpm 20 °C. In lower temperature test experiments, the shaker was inserted into a cooling incubator. The microalgal biomass was heat-pretreated by boiling (5 min at 1 atm) to eliminate the risk of microbial contamination and to ensure controlled conditions except for the harvesting tests performed in wastewater. Microalgae harvesting tests were performed with a fungi:algae mass ratio of 1:2. During each experiment, the concentration of microalgae was measured daily in three repeats to determine the progress of microalgae harvesting. Each parameter was assessed in three independent replicates.

### 2.4. Experimental Harvesting Conditions

First, to identify the fungal species with the most efficient algal recovery capacity, *I. lacteus*, *P. dryinus*, *P. ostreatus*, *T. versicolor*, *P. cinnabarinus* and *T. reesei* were individually added to either *T. obliquus*, *D. communis* or *C. vulgaris* suspensions under the conditions described above.

Secondly, fresh pre-cultured fungal pellets and reused algal-fungal pellets were again added to the respective fresh suspensions of *T. obliquus*, *D. communis* and *C. vulgaris* to determine and compare the efficiency of use of pre-cultured and recycled pellets.

Thirdly, to determine the impact of the bio-flocculation conditions, pH and temperature on the bio-flocculation process, a series of tests were run at a pH from 4 to 9 and at 5, 10, 15 and 20 °C with the same conditions as used previously. For these experiments, only the most efficient fungal species were used.

Lastly, to evaluate the efficiency of the studied microalgae harvesting method under real conditions, primary and secondary wastewater was used as harvesting mediums.

### 2.5. Microalgal Cell Measurements

To determine the reduction in microalgae concentration during the bio-flocculation process, microalgae cell concentration was measured using a UV-visible spectrophotometer (GENESYS 150, Thermo Fisher Scientific Inc., Waltham, MA, USA) at 680 nm absorbance wavelength which is proportional to the change of cell numbers in most unicellular organisms [42]. Microalgae cell concentration in these samples was calculated by measuring the absorption using a UV-visible spectrophotometer in a linear interval. The percentage reduction in the number of microalgae cells in the fluid and, in turn, the harvesting efficiency was calculated using Equation (1):

(1) $$E\%=\frac{C_{0}-\;C}{C_{0}}\times 100\%$$

where E% is the microalgae harvesting efficiency, C_0_ (cell/mL) is the initial microalgae concentration before co-cultivation and C (cell/mL) is the final microalgae concentration in suspension.

Individual calibration curves were constructed for each microalgae strain to relate optical density with the microalgae concentration. Each experiment was performed in three repetitions.

To verify the results obtained with the spectrophotometric method, selected samples of known volume were filtered through a 25 mm diameter 0.2 μm pore–size filter (polycarbonate track-etch membrane, Sartorius, Germany) and stained with 10 μg mL^−1^ DAPI (4′,6-diamidino- 2-phenylindole, Merck, Germany) for 5–10 min according to a protocol described by Denisova et al. (2022) [43]. Cell concentrations were determined with epifluorescence microscopy (Ex: 340/380; Em: > 425, dichromatic mirror 565 nm, Leica DM6000B, Leica Microsystems, Wetzlar, Germany) by counting 20 random fields of view.

### 2.6. Statistical Analysis

Microsoft Excel 2016 *t*-test and ANOVA single-parameter tool (significance level ≤ 0.05) were used for data statistical analysis.

## 3. Results & Discussion

### 3.1. Microalgae Harvesting with Various Fungal Species

Application of white-rot fungi has been recognized in biomass pretreatment, biochemical and enzyme production, biofuel production and bioremediation [44,45,46,47,48,49,50]. Within this study, *Irpex lacteus*, *Pleurotus dryinus*, *Pleurotus ostreatus*, *Trametes versicolor*, *Pycnoporus cinnabarinus* and soil fungus *Trichoderma reesei* were selected as the representative species. First, the highest efficiency of microalgal cell reduction after the addition of fungi was determined. From these, *I. lacteus* provided an average of 98.53 ± 0.36% reduction in microalgae cells after 24 h of harvesting and an average of 99.95 ± 0.05% after 72 h. Further, good harvesting results were obtained with *P. ostreatus*—an average microalgae reduction of 67% was achieved after 24 h, and more than 90% after 48 h. Similar results were obtained after 48 h using *P. dryinus* and *T. versicolor* (85.96 ± 5.74% and 90.53 ± 2.57%, respectively). However, comparing to *I. lacteus*, the efficiency of reduction after 24 h for these fungal species was significantly lower (*p* < 0.05)—47.72 ± 7.22% and 40.54 ± 4.60%, respectively. Bio-flocculation induced by *T. reesei* provided more than 80% microalgae removal efficiency, but after 72 h, an increase in turbidity of the suspension was observed due to active fungal growth.

The only fungal species that did not demonstrate any significant results was *P. cinnabarinus*. The maximum microalgae removal efficiency (38.56 + 9.97%) was obtained after 48 h, following an increase in the turbidity and chromaticity of the suspension. This could be explained by the fact that under certain conditions, *P. cinnabarinus* produces red-to-orange pigmentation due to phenoxazinone pigments, including cinnabarin, tramesanguin and cinnabarinic acid that can be further released in the environment [51].

**Figure 1:**
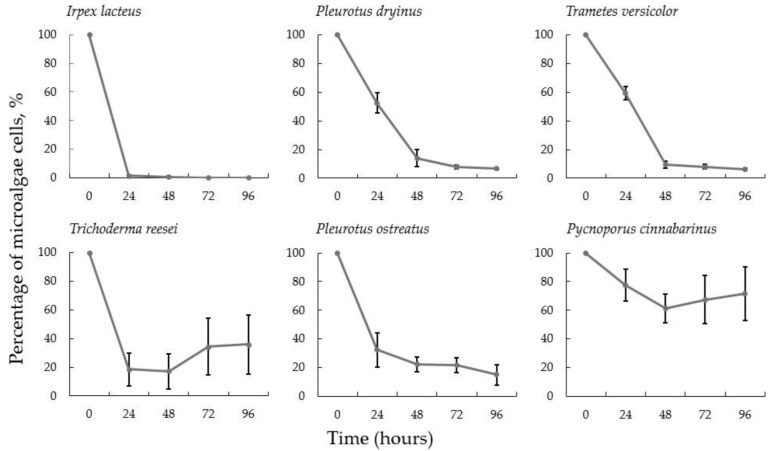
Percentage of suspended *T. obliquus* when selected fungal species were used for bio-flocculation. Standard deviation represents the average from three replicates.

**Figure 2:**
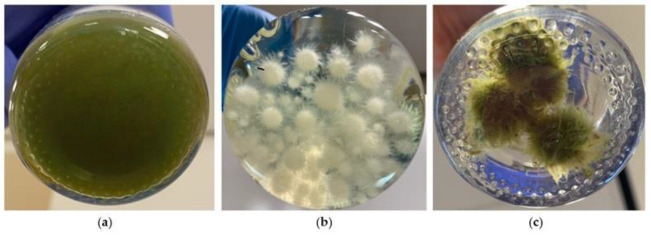
(**a**) *T. obliquus* suspension before bio-flocculation; (**b**) *I. lacteus* culture in liquid medium before adding to the *T. obliquus* suspension; (**c**) algal-fungal pellets formed in *T. obliquus*—*I. lacteus* complex after 24 h.

The obtained results demonstrate that by using white-rot basidiomycetes, in general, more significant bio-flocculation results can be achieved than with *Aspergillus* spp. that typically provide more than 95% after 48 h or longer [21,23,52,53]. Furthermore, *I. lacteus* proved to be the most efficient white-rot fungus for microalgae harvesting (Figure 1) since it showed the most rapid decrease in the suspended microalgae and no algae desorption within 48 h. This could be explained by the fact that *I. lacteus* pellets have more pronounced mycelial filaments compared to other studied fungal species (Figure 2 and Supplementary Materials Figure S1), which potentially increase the surface area of the hyphae and improve sticking.

Further, *I. lacteus* performance was validated with other microalgae, *C. vulgaris* and *D. communis*, since all these species have been applied in tertiary wastewater treatment and demonstrated a high efficiency of phosphorous and nitrogen removal [42,54,55,56,57]. It was reported that *D. communis* is able to ensure 94–100% phosphorous removal [56], and by using *C. vulgaris,* up to 99.8% nitrate and 99.7% phosphate removal can be achieved [54].

**Figure 3:**
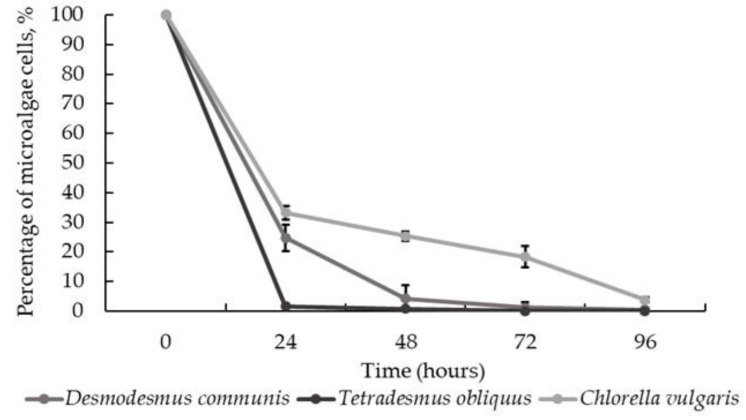
Percentage of selected microalgae remaining in suspension when bio-flocculated with *I. lacteus* against the treatment time. Standard deviation represents the average from three replicates.

In general, more than 90% reduction in all microalgae cells was achieved during the whole treatment time (Figure 3) of 96 h. Twenty-four-h analysis showed a lower reduction efficiency (67.79 ± 2.26%) for *C. vulgaris*. Nevertheless, after 96 h, 96.13 ± 0.90% of *C. vulgaris* was removed. At the same time, it took less than 24 h to harvest up to more than 95% of *T. obliquus* (98.35 ± 0.52%) and 48 h to remove *D. communis* (95.80 ± 4.50%) (Figure 3). The discrepancies in the removal efficiency could be explained by microalgae cell shape differences; however, when taking this into account during the coupling of microalgae assisted tertiary wastewater treatment with *I. lacteus* bio-flocculation technology, the risks of inefficient harvesting can be minimized.

### 3.2. Effect of the Reuse of Algal-Fungal Pellets

Bio-flocculation caused by filamentous fungi can be considered as a potentially environmentally friendly microalgae harvesting technology due to the fact that no addition of toxic chemical coagulants or other chemicals, which are required when using coagulation/flocculation and sedimentation methods, is necessary [14,58]. Thus, by reducing the need of using chemicals in microalgae harvesting, the risk of secondary pollution of wastewater can be reduced. In the case of the studied technology, natural white-rot fungi are used instead of chemicals to induce flocculation of microalgae and to simplify the separation of microalgae cells from the treated wastewater.

**Figure 4:**
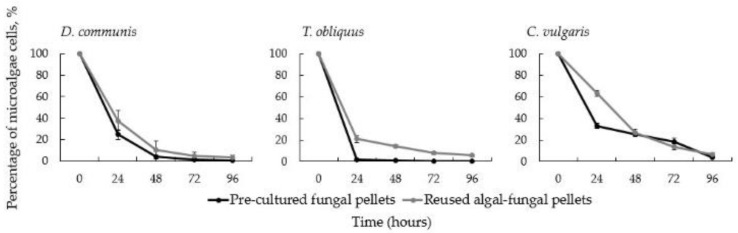
Percentage of selected microalgae remaining in the suspension when pre-cultured *I. lacteus* and recycled algal—*I. lacteus* pellets are used for respective microalgae bio-flocculation. Standard deviation represents the average from three replicates.

It is also essential that the proposed microalgae harvesting technology simultaneously offers the reuse of the harvesting matrix, e.g., algal-fungal pellets in this case. The reuse of fungal pellets can potentially reduce the amount of sludge produced using microalgae harvesting. Thus, the efficiency of using fresh pre-cultured and reused algal-fungal pellets was determined and compared (Figure 4).

As expected, the efficiency of the studied microalgae harvesting method was slightly lower (*p* > 0.05) when used algal-fungal pellets were introduced in the harvesting (Figure 4). Nevertheless, in all cases, the reduction in microalgae cells followed the same pattern and more than 70% after 48 h reduction and more than 80% after 72 h reduction was observed. After 96 h, a reduction of 93.5% (*C. vulgaris*) to 99.95% (*T. obliquus*) was achieved for all three microalgae (Figure 4). Interestingly, a significantly lower harvesting efficiency (*p* < 0.05) of recycled pellets was achieved only after 24 h of *C. vulgaris* bio-flocculation, with an average of 36.86% microalgae reduction (Figure 4). However, despite this fact, the reduction in *C. vulgaris* microalgae after 48 h and longer was practically identical in both cases. In general, a similar trend in the reduction in microalgae concentrations was observed with both pre-cultured and reused pellets.

### 3.3. Impact of Bio-Flocculation Conditions

In order to test the suitability of the investigated technology under practical field conditions, the efficiency of the method was tested at various pH levels and temperatures characteristic for temperate climate zones. Given the role of the pH in the growth of fungi and that changes in pH can affect the surface properties of fungi and the formation of pellets [23], the effect of pH on the bio-flocculation process was investigated and evaluated. It was hypothesized that at a lower pH, bio-flocculation with fungi will be more efficient since acidic conditions are more suitable for fungal growth than microalgae [9]. Furthermore, as the pH of a wastewater can vary, it was necessary to evaluate the impact of this factor on the bio-flocculation process.

**Figure 5:**
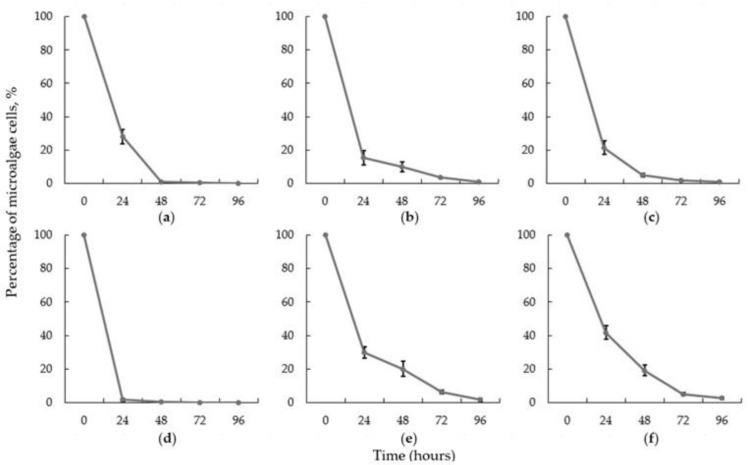
*T. obliquus* harvesting efficiency at pH levels: (**a**) 4; (**b**) 5; (**c**) 6; (**d**) 7; (**e**) 8; (**f**) 9 with *I. lacteus* pellets. Standard deviation represents the average from three replicates.

Here, microalgae co-cultivation with *I. lacteus* was performed at pH 4–9. The highest harvesting rate and efficiency was achieved in the medium with pH 7 (Figure 5). Under neutral conditions, about 99% of the microalgae was harvested within the first 24 h. More than 90% reduction in microalgae was achieved after 48 h at pH 4–6 and after 72 h at a pH above 7. The lowest rate of microalgae removal was observed during bio-flocculation of microalgae at pH 9. This can be explained by the fact that at high pH, net negative charges on microalgae cell walls cause electrostatic repulsion between algal cells, which interferes with the flocculation process [9]. It can be concluded that in the wastewater with a pH around 7, the bio-flocculation process will be most efficient, whereas at a higher or lower pH, it would take longer to ensure the equally high removal of microalgae.

It was determined that the optimal pH conditions for I. lacteus pellets to harvest T. obliquus cells was pH 7. Samples collected from the medium with pH 9 showed the lowest efficiency after 24 h (an average of 62.86 ± 4.08%). However, in general, all results of the T. obliquus harvesting experiments with pH 4.0–9.0 have shown high efficiencies after 72 h—from 94.9 to 99.9% reduction in microalgae cells (Figure 5), so, no significant reduction in the efficiency (p > 0.05) of the technology was observed as a result of the changes in pH. Thus, the technology showed no need for the pH control of the wastewater before microalgae harvesting.

**Figure 6:**
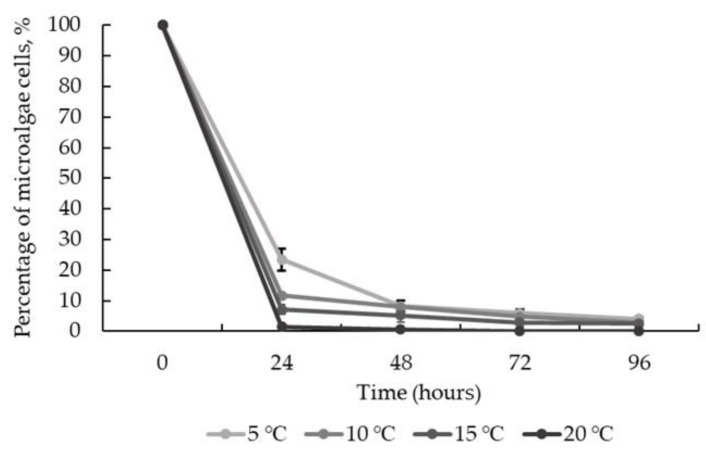
*T. obliquus* harvesting efficiency at 5, 10, 15 and 20 °C with *I. lacteus* pellets. Standard deviation represents the average from three replicates.

To test the possible effect of temperature on the harvesting efficiency, in addition to room temperature incubation, the bio-flocculation process was provided at 5, 10 and 15 °C. In general, after 24 h of incubation, no significant difference (*p* > 0.05) in bio-flocculation efficiency was observed, indicating no effect of temperature on the process. After 48 h, the change in bio-flocculation efficiency was even less significant, with a reduction of 94.84 + 5.16% microalgae concentration at 15 °C, 92.04 + 0.67% at 10 °C and 91.70 + 1.61% at 5 °C. A minor decrease in harvesting efficiency at the initial stage of 5 °C regime was observed; however, prolonged bio-flocculation yielded comparable results under all temperature regimes (Figure 6). Thus, the studied harvesting method is effective even at low temperatures, and after 24 h, 77–93% of microalgae cells are recovered from the liquid.

### 3.4. Algal-Fungal Co-Cultivation in Wastewater

**Figure 7:**
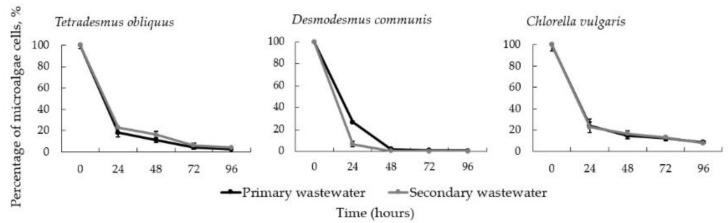
Percentage of *D. communis*, *T. obliquus* and *C. vulgaris* in primary and secondary wastewater after bio-flocculation with *I. lacteus*. Standard deviation represents the average from three replicates.

*D. communis*, *T. obliquus* and *C. vulgaris* suspensions and *I. lacteus* pellets were used in this experiment to test the efficiency of the studied microalgae harvesting method in real wastewater. On average, the concentration of *T. obliquus* was reduced by 82.02 ± 3.05% in the primary wastewater and by 77.19 ± 3.13% in the secondary wastewater after 24 h. *D. communis* was reduced by 73.35 ± 1.10% and 93.22 ± 2.56% in the primary and secondary wastewater, respectively, after 24 h. After the same time, the concentration of C. vulgaris was reduced by 76.12 ± 6.47% in the primary wastewater and by 77.55 ± 5.78% in the secondary wastewater. More than 95% of the *D. communis* microalgae cells were recovered from both wastewater types after 48 h. *T. obliquus* concentration was reduced by more than 90% after 72 h and *C. vulgaris* after 96 h in both wastewater types (Figure 7).

Till now, bio-flocculation of microalgae has been typically associated with the use of filamentous fungi from genus Aspergillus [20,21,22,23,24,25]. Within this study, we have used an alternative approach of replacing Aspergillus with white-rot fungi that have demonstrated their potential in many other biotechnological fields. An especially high effect of bio-flocculation was observed with *I. lacteus*, which is known for its biodegradation ability of dyes, lignocellulose and enzyme production, but has not been previously used for microalgae bio-flocculation from wastewater. Despite the fact that the process efficiency depended on the harvested microalgae species, pH level, temperature and bio-flocculation medium, efficiencies of > 99% can be achieved even with reused pellets in a reasonably short time. Thus, *I. lacteus* can become a good and safe alternative to *Aspergillus* spp. in microalgae bio-flocculation due to its relatively high microalgae-harvesting efficiency and lower risk of negative effects on human health, given that the majority of Aspergillus can be pathogenic [31,32,33]. The lack of processed chemicals and their subsequent removal, recirculation of the fungal pellets and potential further use of the pellets are only some of the positive features of algal—*I. lacteus* complex that allow reduced technological operating costs and environmental sustainability.

## 4. Conclusions

This study confirmed the highly efficient bio-flocculation capacity of *Irpex lacteus* which can provide more than 95% reduction in *Tetradesmus obliquus* and approximately 70% reduction in *Desmodesmus communis* and *Chlorella vulgaris* within 24 h of bio-flocculation at a temperature range from 5–20 °C. In primary and secondary wastewater, 73% to 93% removal efficiency was obtained. Furthermore, *I. lacteus* pellets can be reused for further microalgae harvesting.

Given that no chemicals were needed to induce the flocculation process, the proposed microalgae harvesting method is environmentally friendly, as well as safer for human health and the environment when compared to the use of natural or genetically improved *Aspergillus* spp.

The algal-fungal pellets formed during the bio-flocculation process are characterized by a rather large size (about 5–10 mm) and a high durability. These factors potentially facilitate the removal of microalgae after flocculation, which in turn can also reduce the capital and operational costs of the microalgae harvesting stage. Therefore, the studied technology can potentially become a cost-effective solution for microalgae harvesting. At the same time, the algal-fungal biomass obtained as a result of bio-flocculation could be considered as potential feedstock for the biofuel production. However, further research is needed to identify the effect of white-rot fungi on the harvested biomass composition.

## Figures and Tables

**Table 1:** Parameters of primary and secondary wastewater used for the experiments.

Parameter	Method	Primary Wastewater	Secondary Wastewater	
BOD5 (biochemical oxygen demand)	ISO 5815-1	160	6	mg/L
COD (chemical oxygen demand)	ISO 6060	480	39	mg/L
SS (suspended solids)	EN 872	210	6	mg/L
NH4-N (dissolved ammonium)	ISO 7150-1	45	2.02	mg/L
NO2-N (dissolved nitrite)	ISO 6777:1984 + AC:2001		0.054	mg/L
NO3-N (dissolved nitrate)	ISO 7890-3		3.22	mg/L
PO4-P (dissolved phosphate)	ISO 6878	3.9	0.29	mg/L
pH	ISO 10523	6.9	7.5	

## Supplementary Materials

The following supporting information can be downloaded at: https://www.mdpi.com/article/10.3390/jof8111232/s1, Figure S1: Microalgae harvesting after tertiary wastewater treatment with white-rot fungi
