# Supplementary material for: Microalgae Harvesting after Tertiary Wastewater Treatment with White-Rot Fungi
Source: J Fungi (Basel). 2022 Nov 21;8(11):1232. doi: 10.3390/jof8111232 (PMC9697617; doi:10.3390/jof8111232)
Supplement: Supplementary file 1 [file jof-08-01232-s001.zip › jof-2014031-supplementary.pdf]

Microalgae harvesting after tertiary wastewater treatment with white-rot fungi

Supplementary Figure S1 (**a**) *T. obliquus* suspension before bioflocculation; (**b**) Fungal culture (**1**) *Irpex lacteus*, (**2**) *Pleurotus dryinus*, (**3**) *Pleurotus ostreatus*, (**4**) *Trametes versicolor*, (**5**) *Pycnoporus cinnabarinus*, (**6**) *Trichoderma reesei* in liquid medium before adding to the *T. obliquus* suspension; (**c**) algal-fungal pellets formed in *T.obliquus*-fungi complex after 24 hours.

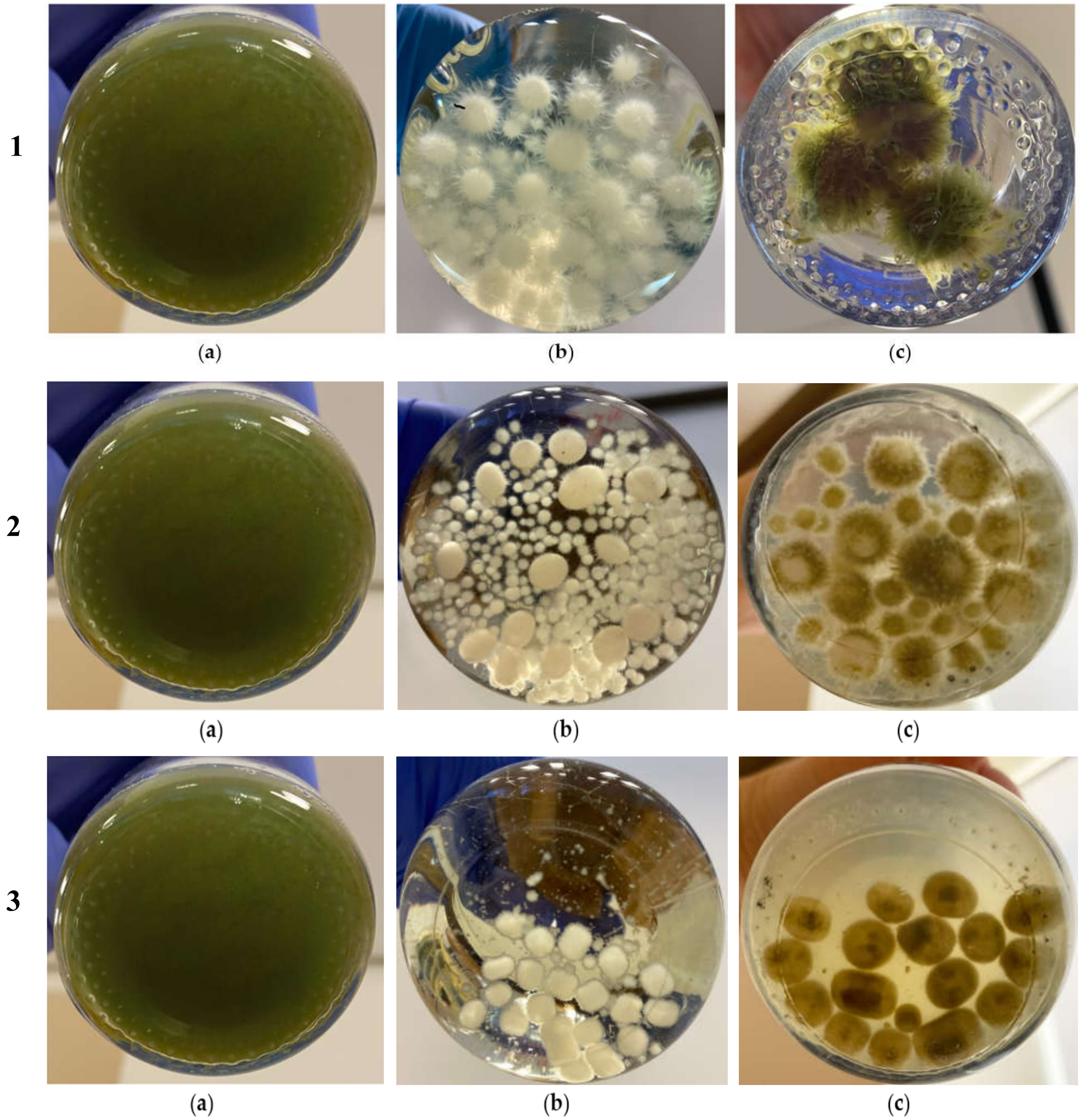

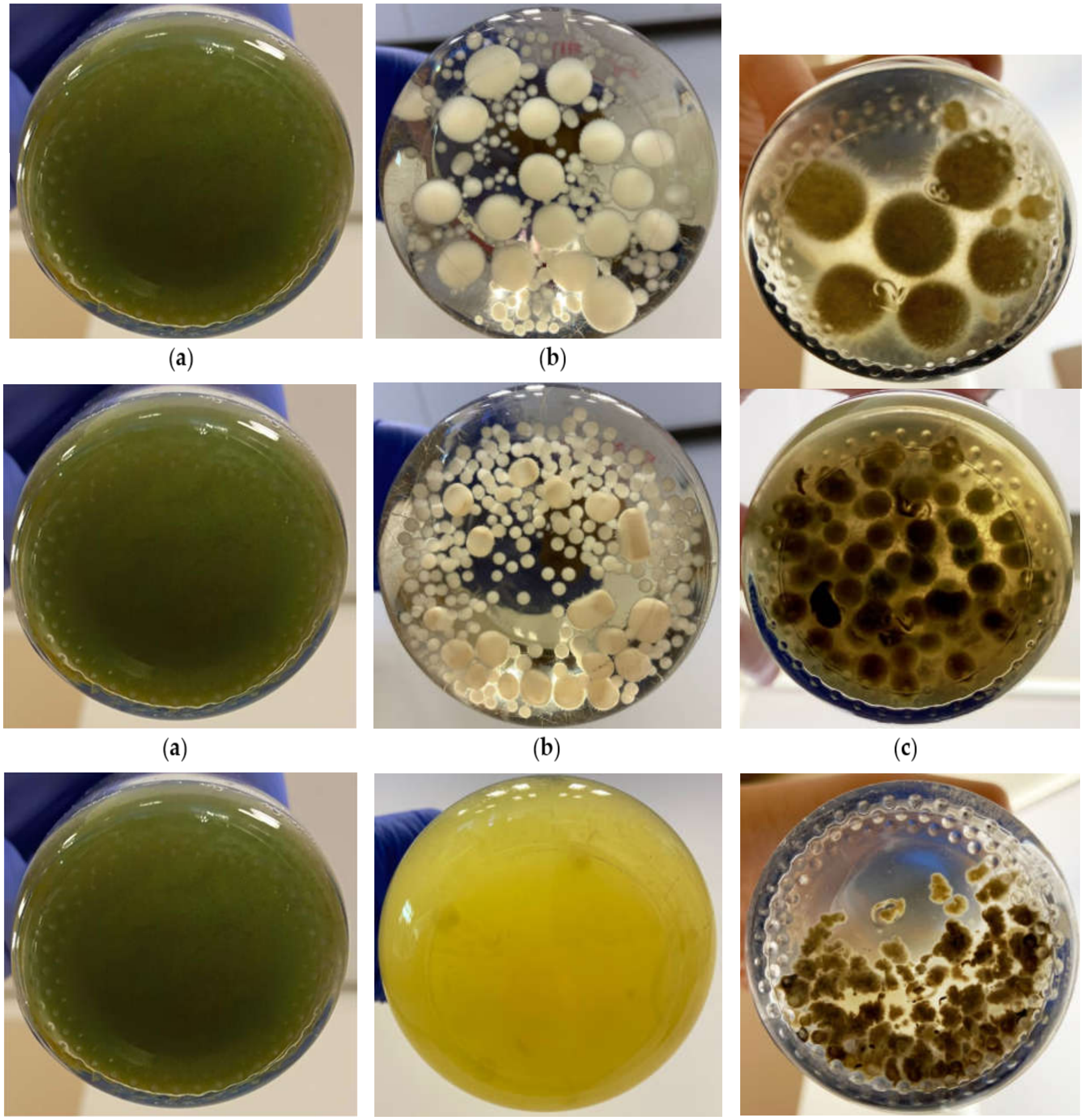

4 (a) (b)

5 (a) (b) (c)

6 (a) (b) (c)
